# Time-Course Changes of Serum Keratin Concentrations after Liver Transplantation: Contrasting Results of Keratin-18 and Keratin-19 Fragments

**DOI:** 10.1155/2020/8895435

**Published:** 2020-11-30

**Authors:** Cristina Macía, Jose Loureiro, Isabel Campos-Varela, Ihab Abdulkader, Esteban Otero, Evaristo Varo, Santiago Tomé, Arturo Gonzalez-Quintela

**Affiliations:** ^1^Department of Internal Medicine and Hepatology, Complejo Hospitalario Universitario de Santiago, Santiago, Spain; ^2^Department of Pathology, Complejo Hospitalario Universitario de Santiago, Santiago, Spain; ^3^Liver Transplant Unit, Complejo Hospitalario Universitario de Santiago, Santiago, Spain

## Abstract

**Objective:**

Under normal conditions, adult hepatocytes express only keratin-8 (K8) and keratin-18 (K18), whereas cholangiocytes also express K19. In this study, we delineate the pattern of normal time-course changes in serum K19 and K18 levels after liver transplantation. *Patients and Methods.* Serum levels of the K19 fragment CYFRA 21-1 and the K18 fragments tissue polypeptide specific antigen (TPS) and M30 (a neoepitope that is generated after caspase cleavage during apoptosis) were measured at baseline and at regular intervals (up to 6 months) after liver transplantation in 11 adult patients.

**Results:**

There was a gradual decrease in serum K19 concentrations from baseline values after transplantation, following a time-course pattern similar to that of serum bilirubin. In contrast, serum concentrations of K18 fragments increased markedly shortly after transplantation and gradually decreased thereafter, following a time-course pattern similar to that of serum transaminases. The increase in TPS tended to occur earlier than that in M30, suggesting an initial predominance of hepatocyte necrosis followed by a predominance of apoptosis in the first days after transplantation. Five patients presented posttransplant complications (acute rejection in three cases and HCV recurrence in two cases). An early increase in serum K19 concentrations was observed in all cases. An increase in serum concentrations of K18 fragments (M30 and TPS) was observed in the two cases with HCV recurrence and was more variable in the three cases with acute rejection.

**Conclusions:**

Serum concentrations of K19 and K18 fragments follow a dissimilar pattern of time-course changes after liver transplantation. The diagnostic value of variations in these normal patterns should be addressed in future studies.

## 1. Introduction

**Figure 1:**
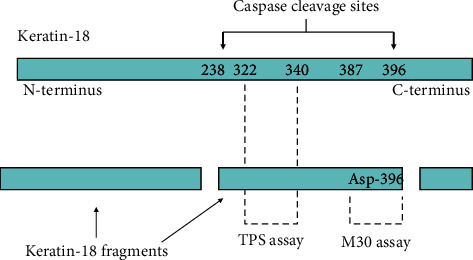
Schematic representation of K18 cleavage during apoptosis and the epitopes recognized by the assays for K18 fragments employed in the present study. The TPS assay can detect soluble full-length K18 as well as K18 fragments; increased serum TPS concentrations could, therefore, represent necrosis or apoptosis. Conversely, the M30 assay detects a neoepitope that is only revealed after caspase cleavage of the protein and is postulated to be a selective biomarker of apoptosis. [13, 16].

Keratins are intermediate filament proteins that are found in epithelial cells [1–6]. They are located intracellularly and are released into the blood upon liver injury, including necrosis and apoptosis [3, 5, 6]. During apoptosis, keratins are substrates for caspase degradation, with subsequent release of keratin fragments to the extracellular space [3, 6, 7]. Serum keratin concentrations are routinely used in clinical practice as tumor markers for epithelial neoplasms [7–9] Immunoassays for detecting keratins can be broadly divided into two types: those that recognize epitopes in intact keratin (or its fragments) and those that recognize specific epitopes generated during apoptosis [6]. Among the former, cytokeratin fragment (CYFRA) 21.1 corresponds to a specific epitope in keratin-19 (K19) [10]. Similarly, tissue polypeptide specific antigen (TPS) and M65 correspond to specific epitopes in K18 [8, 11]. Serum concentrations of all of these keratin fragments increase after epithelial cell necrosis or apoptosis [7, 12]. Among the latter, the M30 assay detects a K18 fragment that includes a neoepitope, Asp396-NE, which is generated after caspase cleavage during apoptosis (Figure 1) [13–15]. The ratio between keratin fragments that increase after apoptosis only and those that increase after either necrosis or apoptosis may offer insights into the relative contribution made by each of these pathologic processes in a given disease [3, 15–17].

Keratins are important proteins in liver health and disease, as reviewed elsewhere [1–6]. Adult hepatocytes express K8 and K18 only [5, 6]. During embryogenesis, hepatocytes also express variable levels of K19, which is also expressed in adult cholangiocytes together with K7 [5, 6]. Keratins are highly abundant in the liver, where they constitute approximately 0.3% of total protein [6]. Their abundance and intracellular distribution make them promising serological markers for nonmalignant liver disease [6]. In fact, serum concentrations of TPS (K18) are increased in most patients with various liver diseases [18, 19], particularly acute hepatitis [19]. Serum TPS concentrations are also markedly increased in patients with steatohepatitis, both alcoholic [19–22] and nonalcoholic [23–25]. In patients with alcoholic liver disease, serum TPS levels serve to distinguish between patients with and without alcoholic steatohepatitis [20]; serum TPS concentrations correlate with the number of Mallory–Denk bodies in liver specimens, a hallmark of steatohepatitis [20,21]. In addition, K18 (M65)-based modification of the MELD score improves the prediction of spontaneous survival after acute liver injury [26]. Serum concentrations of the apoptosis marker M30 have also been extensively studied as a test for liver injury [27–41]. Serum concentrations of the K18 fragment M30 may be useful as a marker for steatohepatitis among patients with either nonalcoholic fatty liver disease [28–30] or alcoholic liver disease [31], as a prognostic marker in alcoholic hepatitis [32], and as a marker for histological severity in patients with chronic hepatitis C [33–35] or hepatitis B [36–38]. Serum M30 levels may also help to differentiate graft-versus-host disease from unrelated conditions with similar liver manifestations in patients who have undergone bone marrow transplantation [39].

Apoptosis is a relevant pathogenetic feature in liver disease [40–43]. It is also a prominent feature after liver transplantation, particularly during ischemia-reperfusion injury [44–46] and rejection [47]. Apoptosis may be a target for therapy in a variety of disorders [48], including liver disease [41, 42, 49]. Antiapoptotic molecules directed at preventing apoptosis during ischemia-reperfusion injury are under investigation [41, 48, 50]. To the best of our knowledge, only two studies have focused on serum keratin levels after liver transplantation. Ulukaya et al. determined the serum concentrations of M65 and M30 up to 24 hours after liver reperfusion [51]. In their study, both apoptosis and, particularly, necrosis were prominent in the short term after liver transplantation from deceased liver donors [51]. Brenner et al. [52] evaluated serum levels of K18 fragments (M65 and M30) after liver reperfusion and during the first days after liver transplantation. Their study also showed an increase of K18, M30, and M65 in the first hours after transplantation, in correspondence with prominent necrosis and apoptosis due to the ischemia/reperfusion injury. Moreover, they also observed an elevation of keratin levels during posttransplant complications such as infection or perfusion disorders [52].

Serum K19 concentrations have been less extensively investigated in patients with liver disease. In a previous study, we found that serum K19 levels are elevated to a lesser extent than K18 levels in patients with alcoholic liver disease [22]. To the best of our knowledge, serum K19 concentrations have not been previously studied in liver transplantation. The objective of the present study was to delineate the normal evolution of serum K19 (CYFRA 21-1) and compare it with that of K18 (TPS and M30) in the midterm (6 months) after transplantation.

## 2. Patients and Methods

### 2.1. Participants and Study Design

This descriptive observational study included 11 consecutive adult patients (aged 40–64 years, seven males) who underwent orthotopic liver transplantation from deceased donors in our centre over a 3-month period. All patients gave written informed consent for participation in the study, which was reviewed and approved by the Institutional Review Board and conformed to the current Helsinki Declaration. The main patient characteristics are presented in Table 1. All of the patients had advanced-stage liver cirrhosis. A history of alcohol abuse was present in eight cases. Standard immunosuppression consisted of corticosteroids and tacrolimus in all participants.

Serum samples for keratin concentration determination were taken at baseline (pretransplantation) and at regular intervals (24 hours, 3 days, 6 days, 10 days, 20 days, 1 month, 2 months, 4 months, and 6 months) after liver transplantation. The serum sample at 24 hours posttransplantation was unavailable for one patient. Standard liver markers were assayed in an ADVIA 1650 analyzer (Bayer Diagnostics, Leverkusen, Germany). Samples were frozen at −40°C until tested for keratin concentrations.

### 2.2. Serum Determinations

#### 2.2.1. Keratin-19 (CYFRA 21-1)

Serum CYFRA 21-1 levels were determined by means of a commercial electrochemiluminescence immunoassay (Elecsys CYFRA 21-1; Roche Diagnostics, Mannheim, Germany) in a Roche Elecsys immunoassay analyzer. The test employs a combination of two monoclonal antibodies (Ks 19.1 and BM 19.21) that are specific to K19 fragments [10]. The epitope sequences recognized by these antibodies lie within the sequence 311–335 for the Ks 19.1 antibody and 346–367 for the detector antibody BM 19.21 [10]. The upper reference limit for serum CYFRA 21-1 is considered to be 3.3 ng/mL.

#### 2.2.2. Keratin-18 (TPS)

Serum TPS concentrations were assayed by means of a commercial chemiluminescent immunoassay (Immulite-TPS; Siemens Medical Solutions Diagnostics, Gwynedd, UK) in an automated platform (Immulite-2000, Siemens). The test employs the M3 monoclonal antibody, which is specific for residues 322–340 in K18 [8, 11] (Figure 1). This method has a detection threshold of 15 U/L. The upper normal reference level was considered to be 100 U/L.

#### 2.2.3. Keratin-18 (M30)

Serum levels of M30 were assayed by means of a commercial immunoassay (M30-Apoptosense; PEVIVA AB, Bromma, Sweden) following the manufacturer's instructions. The test employs the M30 detection antibody, which recognizes a neoepitope mapped to positions 387 to 396 of K18 (Figure 1). This so-called K18-Asp396-NE is only revealed after caspase cleavage of the protein and is postulated to be a selective biomarker of apoptosis [13, 14]. Reference concentrations of M30-antigen were used to calibrate the assay. The absorbance was determined with an ELISA reader at 450 nm. The method has a detection threshold of 25 U/L. The upper normal reference level is considered to be 260 U/L.

### 2.3. Histological Studies

Paraffin-embedded liver specimens were stained with hematoxylin-eosin following standard procedures. For immunohistochemical studies, deparaffinized sections of liver specimens were digested with ChemMate Proteinase K (Dako, Glostrup, Denmark) for 10 minutes prior to antibody incubation. The primary antibody (see below) was applied for 30 minutes at room temperature. The EnVision System (Dako), a dextran polymer that contains peroxidase and the secondary anti-mouse antibody, was then applied for 30 minutes. The sections were washed in the buffer solution (ChemMate Buffer kit, Dako) between incubations. Positive reactions were revealed with diaminobenzidine (DAB, Dako) as the chromogen, and the sections were counterstained with Harris hematoxylin (Panreac, Barcelona, Spain) for 2 minutes. The primary antibodies included K19 (clone RCK 108, Dako), K18 (clone DC10, Dako), and the M30 monoclonal antibody (M30 CytoDEATH; Alexis Biochemicals, Nottingham, UK). The number of hepatocytes containing M30-reactive inclusions per 50 random microscope fields (40x) was considered to be the “apoptotic score” [34, 53].

### 2.4. Statistical Analyses

The Wilcoxon test was employed to compare paired sample values. The Spearman rank test was used to assess correlation. *P* values <0.05 were considered to be statistically significant.

## 3. Results

### 3.1. Evolution of Serum Concentrations of K18 and K19 after Liver Transplantation

**Figure 2:**
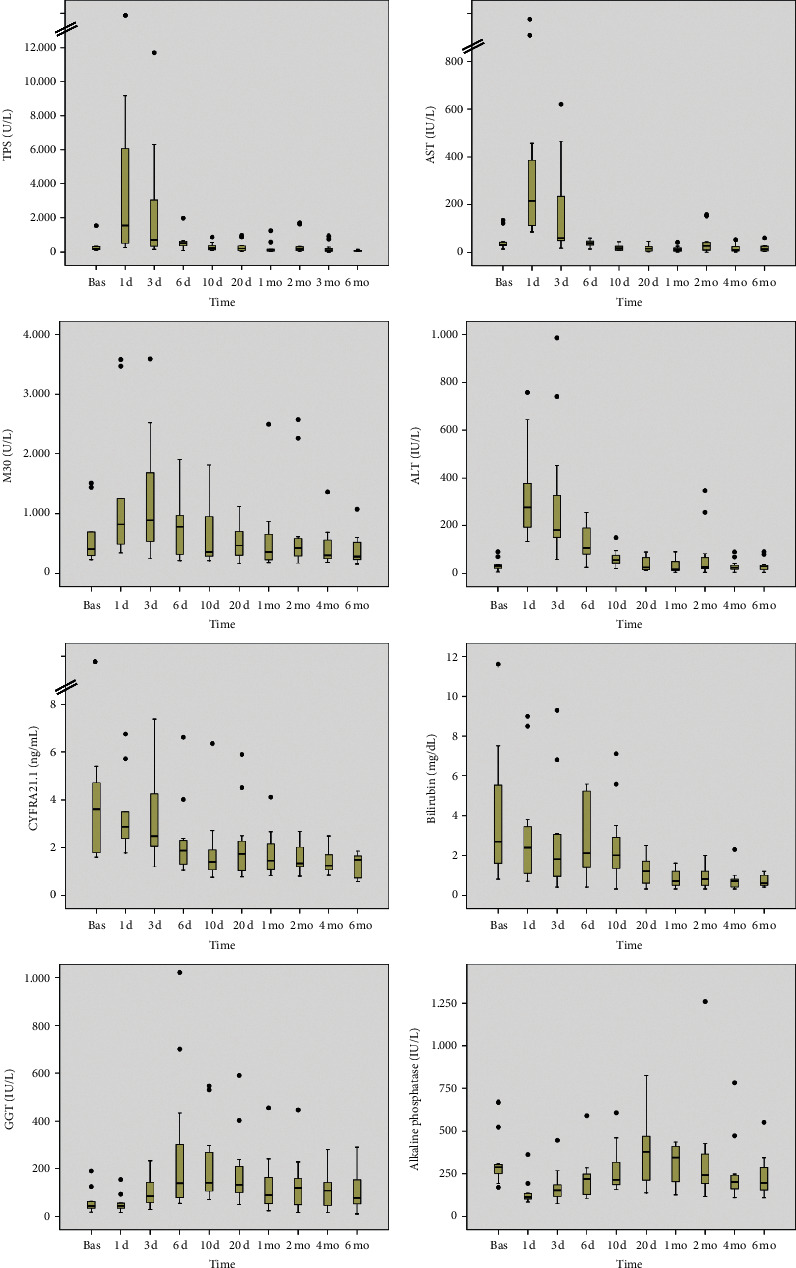
Time-course changes in serum concentrations of the K18 fragments TPS and M30 and the K19 fragment CYFRA 21-1 after liver transplantation. Comparison with time-course changes in the standard liver markers serum AST, ALT, GGT, alkaline phosphatase, and total bilirubin. Bas, baseline (pretransplant level); d, day; mo, month.

**Figure 3:**
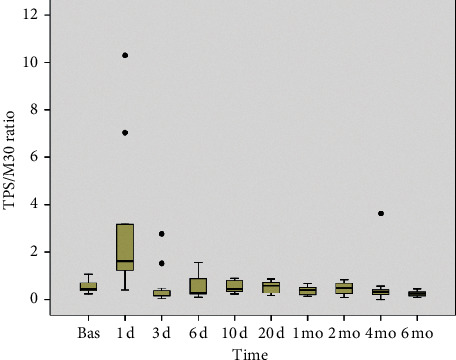
Time-course changes in the ratio between TPS and M30 after liver transplantation.

Pretransplantation levels of CYFRA 21-1, TPS, and M30 were increased in the majority of patients; the median pretransplantation concentrations were 3.6 ng/mL, 201 U/L, and 403 U/L, respectively (Figure 2). Serum concentrations of K18 fragments (TPS and M30) increased dramatically in the immediate posttransplant period (Figure 2). The increase in TPS and M30 occurred in parallel, although in general, the peak TPS concentrations tended to occur earlier (on day +1) than the peak of M30 concentrations, which attained higher median values on day +3 (Figure 2). Consequently, the ratio TPS : M30 in the immediate posttransplant period was highest on day +1 and lowest on day +3 (Figure 3). Serum concentrations of both TPS and M30 decreased thereafter. The decrease in serum TPS concentrations was rather abrupt in most cases, whereas the decrease in M30 concentrations over time was steadier (Figure 2). Six months after transplantation, the serum concentrations of both TPS and M30 were significantly lower than they were in pretransplant samples (Figure 2).

In general, the time-course changes in the concentrations of the K18 fragments TPS and M30 resembled those of the serum transaminases aspartate aminotransferase (AST) and ALT (alanine aminotransferase) (Figure 2). In fact, serum levels of TPS and M30 tended to be correlated with serum levels of AST and ALT. The correlation between K18 fragments and serum transaminases was greater for TPS than for M30 and higher for AST than for ALT, at least in the immediate posttransplant period (Table 2).

Time-course changes in serum K19 (CYFRA 21-1) levels were different from those of K18 fragments. There was no significant peak in CYFRA 21-1 levels immediately after liver transplantation (Figure 2). Instead, there was a steady decrease in the CYFRA 21-1 concentration from pretransplant values over time. From posttransplant day +6 onwards, serum concentrations of CYFRA 21-1 were significantly lower than pretransplantation values (Figure 2). On the whole, the time-course changes in the levels of serum K19 (CYFRA 21-1) resembled those of serum bilirubin (Figure 2). In fact, serum levels of CYFRA 21-1 tended to be correlated with serum bilirubin levels, at least in the immediate posttransplant period (Table 2).

### 3.2. Case Reports

**Figure 4:**
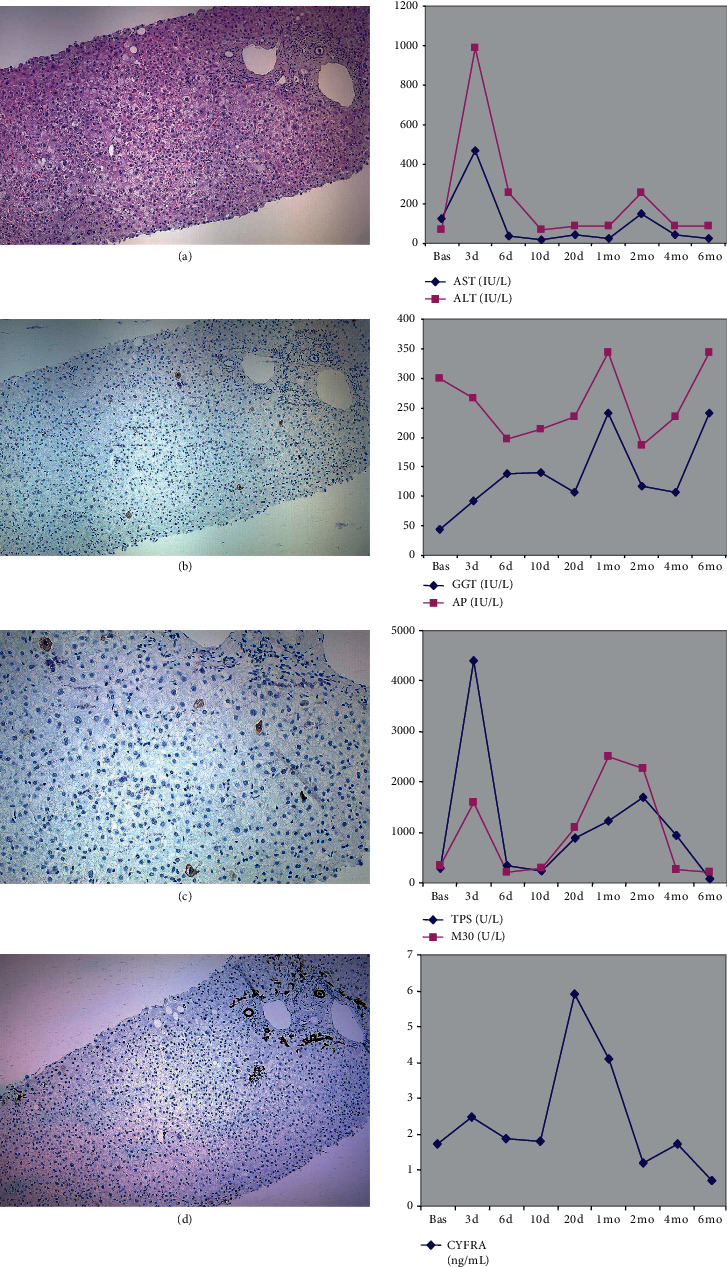
Individual data from Patient 10, who had HCV recurrence after liver transplantation. Liver biopsy (left panels) was performed 3 months after transplantation. (a) Hematoxylin-eosin staining revealed typical signs of HCV recurrence. (b and detail in c) Immunostaining for the M30 neoepitope revealed positivity in apoptotic hepatocytes throughout the liver lobule. (d) Immunostaining for K19 revealed only the normal positivity in bile ducts. Time-course changes in standard biochemical markers (AST, ALT, GGT, and alkaline phosphatase (AP)) and serum keratins (TPS, M30, and CYFRA 21-1) are presented in the right panels. d, day; mo, month.

Posttransplant complications that lead to liver biopsy occurred in five patients (Table 1). Of these, two cases corresponded to recurrent HCV, and the other three corresponded to episodes of acute rejection. Individual data for one of the patients with HCV recurrence are presented in Figure 4. In this case, an increase in serum levels of K18 (TPS and M30) and K19 (CYFRA 21-1) fragments was observed at the time of the recurrence. The increase in serum keratin levels, particularly for K19, tended to occur earlier than alterations in standard liver markers (Figure 4). Liver biopsy was consistent with HCV recurrence (Figure 4). Immunohistochemical studies showed scattered M30-positive hepatocytes throughout the liver lobule (apoptotic score, 17). Immunohistochemical studies in normal control livers were negative (data not shown). Immunohistochemical staining for K19 revealed that the expression of this protein was almost entirely restricted to cholangiocytes (Figure 4). The biochemical and histological findings for the other patient with HCV recurrence were similar (data not shown).

**Figure 5:**
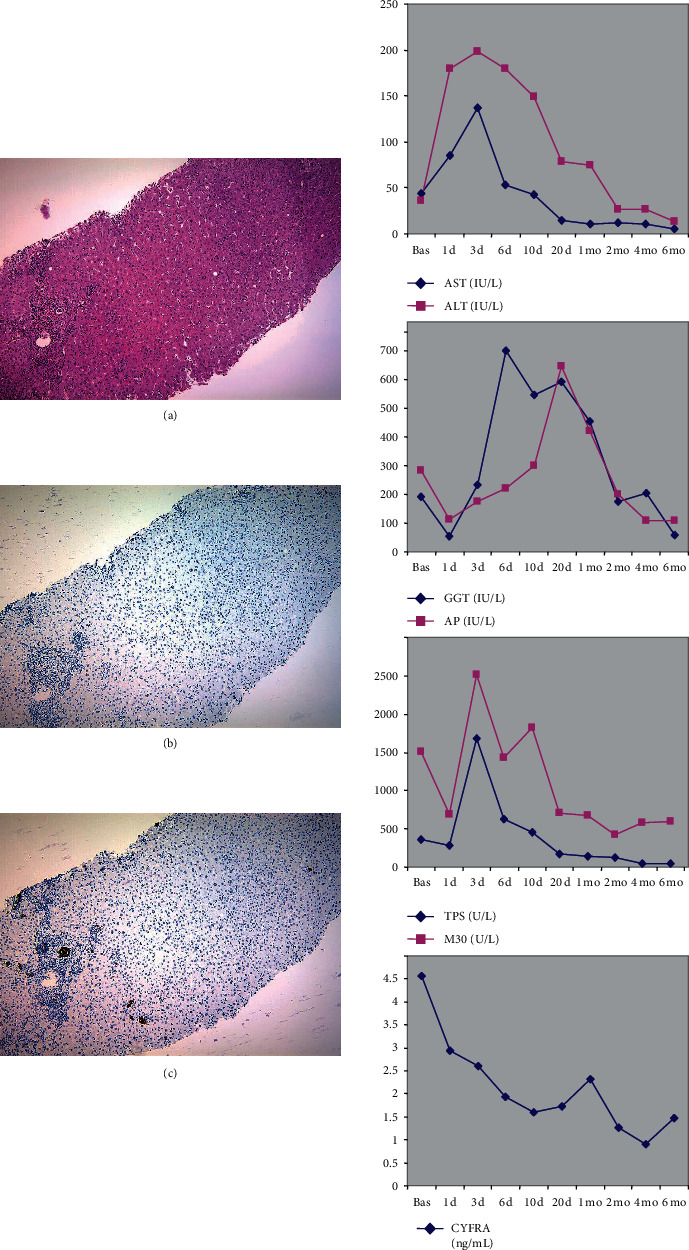
Individual data from Patient 4, who had acute cellular rejection after liver transplantation. Liver biopsy (left panels) was performed on day 33 after transplantation. (a) Hematoxylin-eosin stain revealed typical signs of rejection. (b) Immunostaining for the M30 neoepitope was negative. (c) Immunostaining for K19 revealed the normal positivity in the bile ducts and in a few scattered hepatocytes. Time-course changes in standard biochemical markers (AST, ALT, GGT, and alkaline phosphatase (AP)) and serum keratins (TPS, M30, and CYFRA 21-1) are presented in the right panels. d, day; mo, month.

Individual data for one of the patients with acute rejection are presented in Figure 5. In this case, there was no significant increase in serum K18 fragment (TPS or M30) levels during the episode. Conversely, we observed a transient increase in serum K19 fragment (CYFRA 21-1) (Figure 5). Immunohistochemical studies were negative for M30 (apoptotic score, zero). Immunohistochemical staining for K19 revealed that it was almost entirely restricted to cholangiocytes (Figure 5). For the other two patients with acute rejection (one on day 30 and the other one on day 50), the histological findings were very similar, and biochemical studies also showed a transient increase in K19 fragment (CYFRA 21-1) and a transient increase in K18 fragments (TPS and M30) (data not shown).

## 4. Discussion

Liver transplantation is a standard therapy for advanced liver disease. Improving the understanding of the mechanisms underlying ischemia-reperfusion injury and late complications may serve as a basis for prevention or therapy. In addition, early and accurate detection of the complications that may occur after liver transplantation is of obvious importance. In the present study, we attempted to delineate the normal time-course changes in serum keratins (K19 and K18) in the short and midterm after liver transplantation.

The time-course changes in serum keratin levels indicate that both necrosis and apoptosis of hepatocytes are prominent features immediately after liver transplantation. Serum concentrations of the K18 fragments TPS and M30 increased dramatically in the first 3 days after the procedure. Conversely, serum concentrations of K19 did not increase over the same period. It should be noted that K19 is not constitutively expressed in hepatocytes [1, 3, 6]. The time-course changes in TPS and M30 levels were similar but not strictly parallel. Peak TPS concentrations tended to occur earlier than those of M30. As a consequence, the TPS : M30 ratio was highest on day +1 and lowest on day +3. This indicates that early ischemia-reperfusion liver injury comprises both hepatocyte necrosis and apoptosis, with necrosis predominating in the first hours after transplantation and apoptosis predominating in the following days. This early predominance of hepatocyte necrosis over apoptosis was also reported by Ulukaya et al. [51] and by Brenner et al. [52]. The relative contributions of apoptosis and necrosis could be important for future investigations of methods to prevent early liver dysfunction after transplantation, including the development of antiapoptotic drugs [48, 50]. The increased levels of K18 fragments quickly decreased in the following days, and TPS levels decreased more rapidly than M30 levels. Six months after transplantation, the concentrations of both markers were significantly lower than they were at the pretransplant baseline. Overall, the time-course changes in K18 fragment levels resembled those of the markers of hepatocellular damage such as the serum transaminases AST and ALT. Similar to the results of previous studies, the statistical correlation between K18 fragment concentrations and serum transaminases was higher for AST than for ALT [19].

The time-course changes in K19 fragment (CYFRA 21-1) concentrations after transplantation were entirely different from those of K18 fragments. Serum K19 concentrations showed a steady decrease over time from the pretransplant values, with no significant peak immediately after transplantation. In normal livers, K19 expression is restricted to the biliary epithelium, whereas K18 is, together with K8, the main keratin in hepatocytes [1, 3, 6]. In general, the time-course changes in K19 fragment concentrations resembled those of serum bilirubin.

Regarding the individual case reports, two patients with HCV recurrence showed an increase in serum levels of K18 fragments (TPS and M30), suggesting that there was an increase in necrosis and apoptosis during the episode. Immunohistochemical studies also revealed an enhanced hepatocyte apoptotic score. Interestingly, the increase in serum K18 fragments occurred earlier than that of standard liver markers. Of the three patients with acute cellular rejection, the increase in serum K18 fragments was less evident in one and absent in the other. Furthermore, immunohistochemical studies did not reveal apoptotic hepatocytes in these three cases. Taken together, these findings suggest that apoptosis is more relevant to posttransplant complications such as HCV recurrence than to cellular rejection. Indeed, apoptosis is a relevant feature in chronic hepatitis C [33, 34]. It should be noted that serum levels of K19 fragments conspicuously increased during the episodes of either HCV or acute rejection. In some cases, the K19 increase occurred earlier than that of standard liver markers. Under normal circumstances, K19 is only expressed in biliary epithelial cells, although it can also be expressed in hepatocytes under stress conditions [3, 6]. Bile ducts are well-known targets during both HCV recurrence and acute rejection. In the same way as the changes in K18 levels have been previously related to posttransplant infections and perfusions disorders [52], the typical pattern of time-course changes in K19 levels (a steady decrease from pretransplant values) may make it easier to identify abnormal variations that could indicate posttransplant complications.

The study has limitations that should be acknowledged. It was powered to define the standard pattern of time-course changes in serum keratins. The patients were strictly followed at regular intervals, showing that time-course changes are consistent after transplantation. The study design did not allow us to detect the changes that might have occurred between the scheduled sampling intervals, particularly during the first days after transplantation when the variation was greatest. The sample size was obviously small to evaluate the diagnostic value of serum keratins in this particular setting. Although data from individual case observations should be interpreted with caution, our preliminary results may serve as a basis for further studies of the diagnostic utility of serum keratins for the detection and diagnosis of complications after liver transplantation. It should be noted that keratins are expressed in most simple epithelial cells and are, therefore, not liver-specific [3, 6]. However, the high concentration of keratins in hepatocytes and the time-course changes in their serum concentrations relative to specific events after transplantation strongly indicate that serum keratins measured in our patients originated primarily in the liver.

Measuring the serum levels of keratin fragments may improve our understanding of liver diseases [3, 6]. From a mechanistic standpoint, serum keratins offer a means of measuring necrosis and apoptosis, both of which are important features of liver disease. In this regard, the development of antiapoptotic drugs to prevent early liver transplant-associated liver injury will be of great interest. From a diagnostic standpoint, serum keratin fragments are promising markers of liver disease. For this purpose, key hepatocyte proteins such as keratins may be more robust indicators than standard markers such as enzyme activity, which can be influenced by factors such as drugs in the absence of true liver damage [54]. Assays to determine the concentration of keratin fragments are commercially available, and concentrations may be easily determined with clinically used standard platforms [25]. Here, we have delineated the normal evolution of K18 and K19 fragments after liver transplantation. No conclusions can be drawn from single case reports but may generate discussion to evaluate this biomarker in cases of liver injury, specifically posttransplant complications including rejection. Further studies are warranted to evaluate the utility of keratin fragments as markers of disease in liver transplant patients.

## Figures and Tables

**Table 1:** Patient characteristics.

Patient number	Age (y)/sex	Indication for transplantation	Ischemia time (min)	Complications after transplantation
1	56/M	Cirrhosis (alcohol plus HCV)	260	Recurrent HCV hepatitis
2	56/F	Cirrhosis (alcohol) and hepatocellular carcinoma	720	None
3	45/F	Cirrhosis (alcohol)	690	*De novo* diabetes mellitus
4	43/M	Cirrhosis (alcohol)	360	Acute cellular rejection (day +33); *de novo* diabetes mellitus
5	53/M	Cirrhosis (alcohol)	700	Acute cellular rejection (day +10)
6	40/F	Cirrhosis (alcohol)	240	Intestinal obstruction (umbilical hernia)
7	62/F	Cirrhosis (autoimmune)	420	None
8	58/M	Cirrhosis (alcohol)	300	Mild kidney failure
9	55/M	Cirrhosis (HBV) and hepatocellular carcinoma	630	None
10	64/M	Cirrhosis (HCV)	360	Recurrent HCV hepatitis
11	61/M	Cirrhosis (alcohol)	300	Acute cellular rejection (day +56); *de novo* diabetes mellitus; mild kidney failure

HBV, hepatitis B virus; HCV, hepatitis C virus. The initial immunosuppression regime included tacrolimus and corticosteroids in all cases.

**Table 2:** Correlation between serum keratin concentrations and standard liver markers in the immediate posttransplant period.

	Day +1			Day +3			Day +6		
	AST (IU/L)	ALT (IU/L)	Bilirubin (mg/dL)	AST (IU/L)	ALT (IU/L)	Bilirubin (mg/dL)	AST (IU/L)	ALT (IU/L)	Bilirubin (mg/dL)
CYFRA 21-1 (U/L)	0.273	0.152	0.681^*∗*^	0.618^*∗*^	0.409	0.191	0.451	0.183	0.781^*∗∗*^
TPS (U/L)	0.855^*∗∗*^	0.818^*∗∗*^	0.401	0.900^*∗∗*^	0.818^*∗∗*^	0.182	0.724^*∗∗*^	0.320	0.603^*∗*^
M30 (U/L)	0.661^*∗*^	0.697^*∗*^	0.602	0.691^*∗*^	0.655^*∗*^	0.382	0.287	0.078	0.461

CYFRA 21-1, keratin-19 fragment; TPS, tissue polypeptide specific antigen (keratin-18); M30, neoepitope generated during apoptosis in caspase-cleaved keratin-18; AST, aspartate aminotransferase; ALT, alanine aminotransferase.^*∗*^*P* < 0.05 and ^*∗∗*^*P* < 0.005.

## Data Availability

The data used to support the findings of this study are available from the corresponding author upon request.
